# NLRP3-Inflammasome Activating DAMPs Stimulate an Inflammatory Response in Glia in the Absence of Priming Which Contributes to Brain Inflammation after Injury

**DOI:** 10.3389/fimmu.2012.00288

**Published:** 2012-09-18

**Authors:** Catherine Diane Savage, Gloria Lopez-Castejon, Adam Denes, David Brough

**Affiliations:** ^1^Faculty of Life Sciences, University of ManchesterManchester, UK

**Keywords:** inflammation, caspase-1, priming, interleukin-1, NLRP3-inflammasome, cerebral ischemia

## Abstract

Inflammation in the absence of infection (sterile inflammation) contributes to acute injury and chronic disease. Cerebral ischemia is a devastating condition in which the primary injury is caused by reduced blood supply and is therefore sterile. The cytokine interleukin-1β (IL-1β) is a key contributor to ischemic brain injury and central inflammatory responses. The release of IL-1β is regulated by the protease caspase-1, and its activating complex, the inflammasome. Of the known inflammasomes the best characterized, and one that is perceived to sense sterile injury is formed by a pattern recognition receptor called NOD-like receptor pyrin domain containing three (NLRP3). A key feature of NLRP3-inflammasome dependent responses *in vitro* in macrophages is the requirement of an initial priming stimulus by a pathogen (PAMP), or damage associated molecular pattern (DAMP) respectively. We sought to determine the inflammatory responses of NLRP3-activating DAMPs on brain derived mixed glial cells in the absence of an initial priming stimulus *in vitro*. In cultured mouse mixed glia the DAMPs ATP, monosodium urate, and calcium pyrophosphate dehydrate crystals had no effect on the expression of IL-1α or IL-1β and induced release only when the cells were primed with a PAMP. In the absence of priming, these DAMPs did however induce inflammation *via* the production of IL-6 and CXCL1, and the release of the lysosomal protease cathepsin B. Furthermore, the acute phase protein serum amyloid A (SAA) acted as a priming stimulus on glial cells resulting in levels of IL-1 expression comparable to those induced by the PAMP lipopolysaccharide. *In vivo*, after cerebral ischemia, IL-1 production contributed to increased IL-6 and CXCL1 since these cytokines were profoundly reduced in the ischemic hemispheres from IL-1α/β double KO mice, although injury-induced cytokine responses were not abolished. Thus, DAMPs augment brain inflammation by directly stimulating production of glial derived inflammatory mediators. This is markedly enhanced by DAMP-induced IL-1-release-dependent responses that require a sterile endogenous priming stimulus such as SAA.

## Introduction

Interleukin-1β (IL-1β) is a key pro-inflammatory cytokine that is central to the damaging inflammatory processes that accompany sterile disease (Dinarello, 2011). This is particularly true after an acute brain injury such as cerebral ischemia, or stroke, where IL-1β is established as a major contributor to damage (Brough et al., 2011). It is produced during disease or after an injury as an inactive precursor (pro-IL-1β) by cells of the innate immune system such as macrophages, or in diseases of the central nervous system (CNS), by microglia (Denes et al., 2008). In order for it to exert any biological effects it must be cleaved into an active molecule and released from the cell whereby it can act on the type I IL-1 receptor (IL-1RI) on responsive cells (Luheshi et al., 2009). A key protease required for the processing of pro-IL-1β is caspase-1. The activity of caspase-1 is regulated by its recruitment to multi-molecular scaffolds called inflammasomes following an inflammatory stress (Schroder and Tschopp, 2010). Inflammasomes are composed of a cytosolic pattern recognition receptor (PRR), pro-caspase-1, and, depending on the PRR, an adaptor molecule. The best characterized inflammasome forming PRR, and the one most implicated as a sensor of sterile injury, is NOD-like receptor pyrin domain containing three (NLRP3; Cassel and Sutterwala, 2010; Schroder and Tschopp, 2010). NLRP3 can be activated by a diverse array of disease associated molecules, where it oligomerizes with the adaptor ASC (apoptosis-associated speck-like protein containing a caspase recruitment domain) and caspase-1 to form the NLRP3-inflammasome, resulting in the processing of pro- to mature IL-1β and its release.

The release of IL-1β is considered to be a two step process (Hornung and Latz, 2010; Lopez-Castejon and Brough, 2011). IL-1β is not normally expressed and so its expression must be induced. Stimuli that do this are pathogen associated molecular patterns (PAMPs) or damage associated molecular patterns (DAMPs; Chen and Nunez, 2010; Takeuchi and Akira, 2010). PAMPs are motifs carried by pathogens, such as bacterial endotoxin (or lipopolysaccharide, LPS) of Gram negative bacteria, and DAMPs are endogenous molecules modified during disease or that are released by necrosis. Stimuli that prime macrophages in this way are however generally inefficient secretion stimuli for IL-1β and the primed cells are required to encounter an additional PAMP or DAMP stimulus that triggers formation of the inflammasome, activation of caspase-1, and subsequently the processing and secretion of IL-1β. This comes from an extensive literature on macrophages, and although there is evidence that microglia respond in a similar way to PAMP and DAMP stimulation (Brough et al., 2002; Halle et al., 2008), these responses have yet to be fully characterized. Not only are microglia a unique cell type originating from the yolk sac that are self-renewing throughout life (Ginhoux et al., 2010), but the brain is also protected by the BBB in the absence of injury, which keeps microglia isolated from potential blood-derived priming stimuli. In addition, inflammatory events triggered by DAMPs, other than IL-1 expression or release, have not been explored in glial cells previously.

The effects of NLRP3-activating DAMPs are commonly associated with IL-1 and on primed cells (Hornung and Latz, 2010). This is at least in part because NLRP3 expression is itself dependent upon priming (Bauernfeind et al., 2009). However, in many situations of acute injury, where there is rapid and localized loss of tissue (such as cerebral ischemia for example) there will be an abundance of DAMPs that may stimulate cells that have not been subject to an initial priming stimulus. LPS (often used to prime cells *in vitro*) is unlikely to be present *in vivo* during sterile injury and endogenous priming stimuli in the brain remain poorly characterized. DAMPs such as monosodium urate (MSU), and calcium pyrophosphate dehydrate (CPPD) crystals induce the production of the pro-inflammatory cytokine IL-6 in monocytes (Guerne et al., 1989) and osteoblast like cells (Bouchard et al., 2002) in the absence of a priming stimulus. MSU is also known to act as a potent adjuvant in driving adaptive immune responses independently of the NLRP3-inflammasome (Kool et al., 2011) and potentially dependent upon Syk kinase (Ng et al., 2008). Inflammatory responses in the brain (after cerebral ischemia for example) may be influenced by both local DAMPs and circulating inflammatory mediators once the breakdown of the BBB has occurred. As acute brain injury is associated with a marked central inflammatory response the aim of this research was to identify the inflammatory responses of glial cells, inflammatory cells of the CNS, to key mediators of sterile injury, NLRP3-activating DAMPs, in the absence of cell priming, and in the presence of a relevant endogenous priming stimulus.

## Materials and Methods

### Materials

DMEM culture media was purchased from Sigma (UK). Fetal bovine serum (FBS), glutamine, and a streptomycin/penicillin antibiotic solution were all purchased from Invitrogen (UK). Bacterial LPS (*Escherichia coli* 026:B6), Poly(IC), and ATP were purchased from Sigma (UK). MSU and CPPD crystals were from Invivogen (UK). Serum amyloid A (SAA) was purchased from PeproTech (UK). All primers for qPCR were purchased from Qiagen (UK).

### Middle cerebral artery occlusion, perfusion, and tissue homogenization

We induced cerebral ischemia by middle cerebral artery occlusion (MCAo) as described previously (Denes et al., 2010a). Briefly, C57BL/6J mice (Harlan Olac) or IL-1αβ-deficient (IL-1αβ double KO) mice, weighing 26–32 g were anesthetized with isoflurane and were subjected to MCAo for 60 min using an intraluminal filament (180 μm diameter, left side occluded) followed by 24 h reperfusion. After transcardial perfusion with saline, brains were collected, and homogenized as described previously (Chapman et al., 2009). Protein concentrations were calculated using BCA assay (Pierce/Thermo Fisher Scientific). Some mice were perfused with 4% paraformaldehyde (PFA), and following post-fixation in PFA and cryoprotection in sucrose, brain sections were cut on a sledge microtome for immunohistochemistry and cresyl-violet staining. All animal procedures were performed under the University of Manchester project license number (40/3076) and adhered to the UK Animals (Scientific Procedures) Act (1986).

### Immunohistochemistry

Immunostaining was performed on free-floating brain sections as described (Denes et al., 2010a). After blocking with 2% normal donkey serum in PBS containing 0.3% Triton X-100, rabbit anti-Iba1 (WAKO, Germany) and rat anti-CD45 (Serotec, UK) antibodies were incubated overnight. Antigens were visualized using appropriate fluorochrome (Alexa 594, Alexa 488)-conjugated donkey secondary antibodies (Invitrogen). Mounted brain sections were coverslipped with ProLong mounting medium (Invitrogen) and analyzed on an Olympus BX51 microscope using a Coolsnap ES camera (Photometrics) through MetaVue software (Molecular Devices). CD45 and Iba 1 positive cells were quantified by counting six separate sections from the ipsilateral and contralateral hemispheres (striatum and cortex). The numbers of Iba 1 positive cells expressing CD45 were recorded for both hemispheres.

### Cell culture

Mixed glia were cultured from C57Bl/6J at post-natal day 1–4 as described previously (Pinteaux et al., 2002). Whole brains were dissected into DMEM with 10% FBS v/v and 1% P/S. Meninges were removed and cells dissociated by trituration prior to seeding at a density equivalent to one brain/60 cm^2^. The culture medium was changed twice a week until cultures reached confluency (14–20 days). These cultures are composed of 78% astrocytes, 12% O2A progenitor cells, and 10% microglia (Pinteaux et al., 2002). Cultures were treated with LPS (1 μg/ml), poly(IC; 50 μg/ml), ATP (5 mM), MSU (250 μg/ml), CPPD (250 μg/ml), or SAA (0.03–3 μg/ml) for 24 h. Cultures subjected to PAMP and DAMP stimulation were treated with LPS or SAA for 24 h followed by ATP, MSU, or CPPD for 1 h.

### Quantitative real-time PCR

RNA was extracted from cultured mixed glia using the TRIzol^®^ method (Invitrogen) and reverse transcribed to cDNA using MMLV reverse transcriptase according to the manufacturer’s instructions (Invitrogen). Specific primers for IL-1β, IL-1α, caspase-1, NLRP3, ASC, iNOS, IL-6, TNFα, CXCL1, and CXCL12 were purchased from Qiagen (QuantiTech Primer Assays) and qPCR was performed using Power SYBR^®^ Green PCR mastermix (Applied Biosystems) and SDS v2.3 (Applied Biosystems). For each primer set the CT threshold was set manually to achieve a slope efficiency of >99% and a single product on melt curve analysis. RNA from LPS-treated J774 macrophages was used to create a standard curve and gene expression was calculated using the relative standard curve method. Data were normalized to expression levels of the housekeeping gene SDHA (QuantiTech Primer Assays, Qiagen) across each treatment and fold change was expressed relative to basal RNA levels from untreated mixed glia.

### Detection of cytokines by ELISA

Measurement of key inflammatory cytokines (IL-1β, IL-1α, IL-6, CXCL1) released into the culture supernatant or expressed in the lysate was performed using specific ELISAs (R&D Systems, UK) according to manufacturers guidelines.

### Detection of cytokines by ELISA and cytometric bead array

Measurement of cytokines released into the culture supernatant or expressed in the lysate was performed using specific ELISAs (R&D Systems, UK) according to manufacturer’s guidelines. Key inflammatory cytokines (IL-1β, IL-1α, IL-6, CXCL1) were measured in all tissues examined using appropriate cytometric bead array (CBA) Flex Sets (BD Biosciences) according to the manufacturer’s protocol.

### Flow cytometric analysis

Cultured mixed glia were resuspended using 0.5 mM EDTA in PBS. The following fluorochrome-labeled monoclonal antibodies were applied according to manufacturer’s instructions: PE conjugated anti-CD11c (1:100, eBioscience), APC conjugated anti-MHCII (1:200, eBioscience), FITC conjugated anti-CD11b (1:200, eBioscience), and PerCP-Cy5.5 conjugated anti-CD45 (1:500, eBioscience). The surface expression of these markers were analyzed using CyAn advanced flow cytometer (Beckman Coulter) and Summit v4.3 software (Dako). Microglia were gated for analysis based on co-expression of CD11b and CD45.

### Western blotting

Following the experiment supernatants were harvested and prepared in sample buffer containing 1% β-mercaptoethanol. Samples were boiled and then electrophoresed on 12% SDS-acrylamide gels. Proteins were subsequently transferred onto nitrocellulose membrane and blotted using polyclonal sheep anti-mouse IL-1β (1:1000 in 5% milk, NIBSC, UK), or polyclonal goat anti-mouse cathepsin B (1:500, R&D Systems). The membrane was then stained with polyclonal rabbit anti-sheep IgG horse radish peroxidise (HRP) conjugate for IL-1β (1:2000 in 5% milk, Dako, UK), or with HRP-conjugated rabbit anti-goat IgG for cathepsin B (1:1000 in 5% milk, Dako, UK), with subsequent exposure using enhanced chemi-luminescence (ECL) reagents (Amersham, UK).

### Gel zymography

Released gelatinase activity was assessed by gelatin-substrate zymography as previously described (Kleiner and Stetler-Stevenson, 1994). Briefly, serum free supernatants from treated cultured mixed glia were mixed with an equal volume of loading buffer (10% SDS, 50% glycerol, 400 mM Tris-HCl pH 6.8, 250 μg/ml bromophenol blue). All samples were loaded neat onto 8% zymography gels except for one of the ATP-treated supernatants (+*) which was diluted 1:3. Following electrophoresis, gels were washed in 2.5% Triton X-100 to remove SDS. Proteinases were renatured in activity buffer (50 mM Tris-HCl pH 7.5, 5 mM CaCl_2_, 5 μM ZnCl_2_, 0.02% NaN_3_) for 96 h at 37°C prior to staining in 0.5% Coomassie Brilliant Blue R-250 in 40% methanol and 10% acetic acid for 1 h at rt. Gels were destained at rt in 10% acetic acid, 10% methanol until clear bands appeared. Molecular weight of bands was estimated against molecular weight markers (BioRad).

### Data analysis

All quantitative assessments were performed in a blinded manner. Unless stated otherwise, for two groups paired *t*-test (two-tailed), for three or more groups one-way or two-way analysis of variance (ANOVA) followed by Bonferonni’s *post hoc* multiple- or paired-comparison were used. All data are expressed as mean ± SD. ***P < 0.001, **P < 0.01, *P < 0.05.

## Results

**Figure 1:**
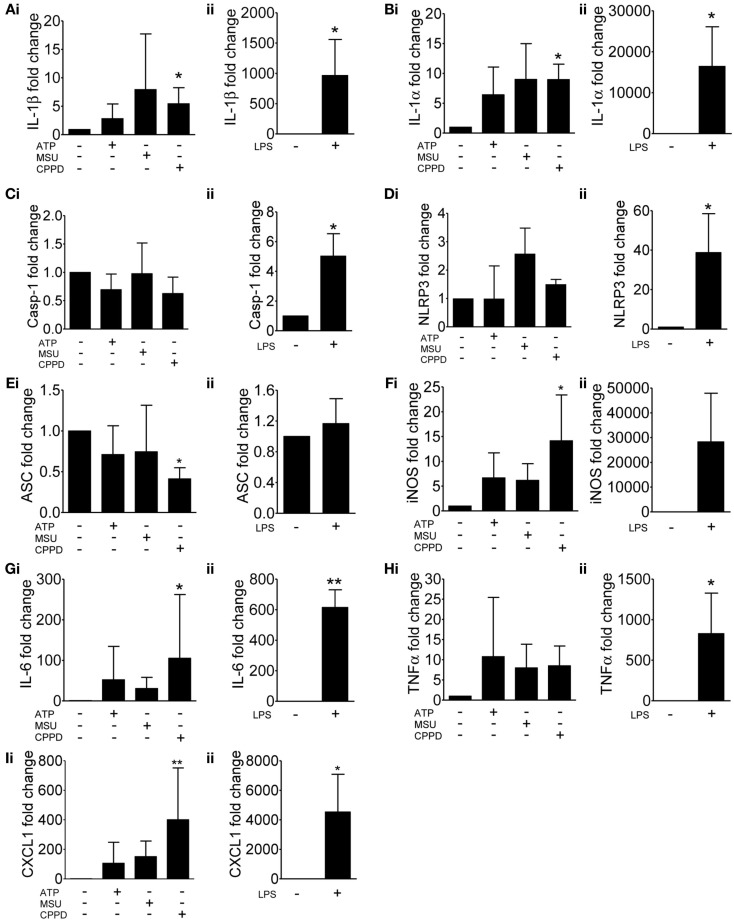
**Effects of DAMPs on the expression of pro-inflammatory genes in cultured mixed glia**. mRNA levels of pro-inflammatory genes were measured by qPCR after 4 h exposure to the NLRP3-activating DAMPs ATP (5 mM), MSU, and CPPD (both 250 μg/ml) **(Ai)** or the PAMP LPS (1 μg/ml) **(Aii)**. Data were normalized to expression levels of the housekeeping gene SDHA across each treatment and fold change was expressed relative to basal RNA levels from untreated mixed glia. The genes analyzed were IL-1β **(A)**, IL-1α **(B)**, caspase-1 **(C)**, NLRP3 **(D)**, ASC **(E)**, iNOS **(F)**, IL-6 **(G)**, TNFα **(H)**, and CXCL1 **(I)**. Data are pooled samples from at least five separate experiments. **P < 0.01, *P < 0.05, vs. untreated.

As discussed above, NLRP3 is proposed as a sensor of sterile injury and disease and recognizes a wide range of structurally diverse DAMPs (Cassel and Sutterwala, 2010). We selected three of the best characterized NLRP3-activating DAMPs for our investigation into the effects of cell priming. ATP activates NLRP3 *via* its activation of cell surface P2X7 receptors (Mariathasan et al., 2006). MSU and CPPD crystals, inflammatory drivers of gout, and pseudogout respectively also activate the NLRP3-inflammasome (Martinon et al., 2006). Uric acid crystals are also suggested to be a general inflammatory signal released from dead and dying cells (Kono et al., 2010). We initially looked at the effects of these NLRP3-activating DAMPs on cell priming itself. Primary cultures of mouse mixed glia (composed of astrocytes and microglia; Pinteaux et al., 2002) were treated with ATP, MSU, or CPPD crystals for 4 h after which lysates were harvested and analyzed by qPCR for the expression of markers of inflammatory cell priming (Figure 1). As a positive control cultures were treated with the PAMP LPS. We initially investigated the effects of these DAMPs and LPS on the expression of genes typically associated with the inflammasome and priming e.g., IL-1β (Figure 1Ai), IL-1α (Figure 1Bi), caspase-1 (Figure 1Ci), NLRP3 (Figure 1Di), and ASC (Figure 1Ei). In general, DAMPs had no effect on the expression of these genes except for CPPD crystals, where a significant increase in the expression of IL-1β and IL-1α was observed (Figures 1Ai,Bi). LPS stimulation increased the expression of all genes, including caspase-1 (Figure 1C) and NLRP3 (Figure 1D), but did not affect ASC, whose expression did not change with any treatment except with CPPD crystals where a significant decrease was observed (Figure 1E).

In order to determine whether these NLRP3-activating DAMPs were capable of stimulating a more general inflammatory response we extended our study on gene expression to include additional inflammatory genes such as iNOS (Figure 1F), IL-6 (Figure 1G), TNFα (Figure 1H), and CXCL1 (Figure 1I). Again, DAMPs had no effect, with the exception of CPPD crystals on the expression of iNOS (Figure 1F), IL-6 (Figure 1G), and CXCL1 (Figure 1I), whilst LPS induced a robust response across all genes tested. These data suggest that at the level of gene expression, and within the limits of the genes investigated in this study, and with the exception of CPPD, NLRP3-activating DAMPs have a negligible effect.

**Figure 2:**
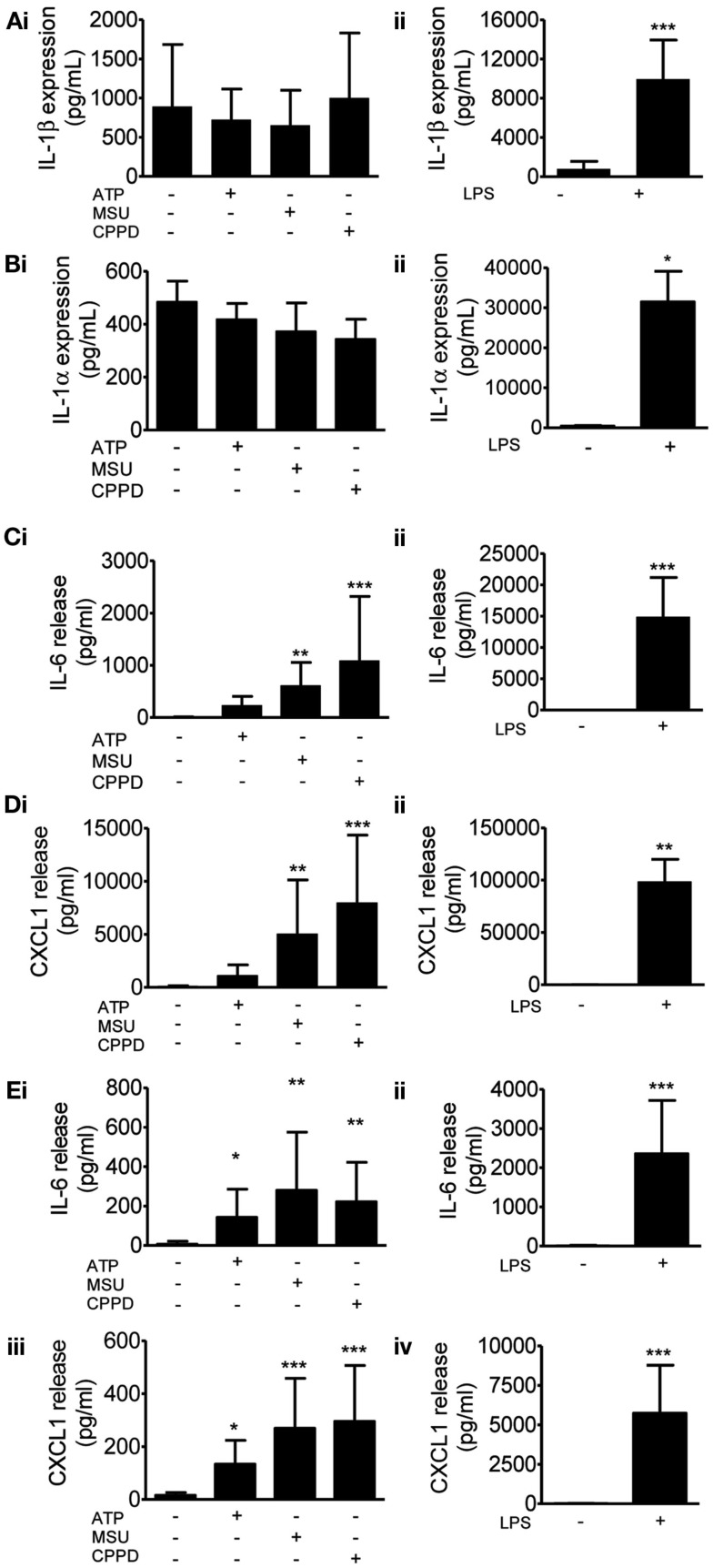
**Effects of DAMPs on pro-inflammatory protein levels in cultured mixed glia**. Protein levels of pro-inflammatory mediators were measured by specific ELISAs after 24 h **(A–D)** or 4 h **(E)** exposure to the NLRP3-activating DAMPs ATP (5 mM), MSU, and CPPD [both 250 μg/ml; **(Ai)**] or the PAMP LPS [1 μg/ml; **(Aii)**]. The proteins analyzed were IL-1β **(A)**, IL-1α **(B)**, IL-6 **(C,E)**, CXCL1 **(D,E)**. Data are pooled samples from at least five separate experiments. ***P < 0.001, **P < 0.01, *P < 0.05, vs. untreated.

**Figure 3:**
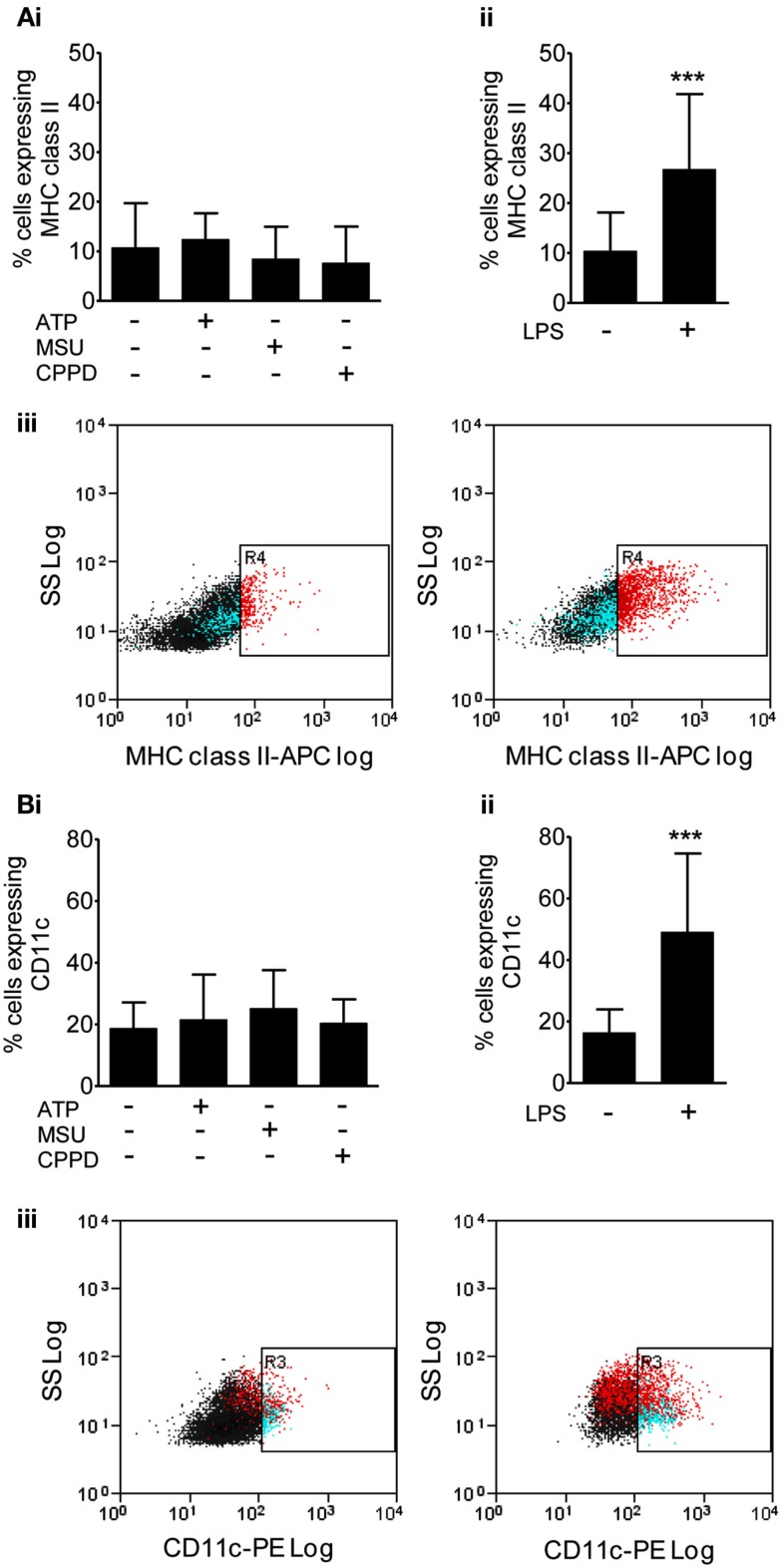
**Effects of DAMPs on surface marker expression in microglia cells**. The expression of surface markers of microglia activation MHC class II **(A)**, and CD11c **(B)** were measured after 24 h exposure to the NLRP3-activating DAMPs ATP (5 mM), MSU, and CPPD [both 250 μg/ml; **(Bi)**] or the PAMP LPS [1 μg/ml; **(Bii)**]. Flow cytometry was used to quantify surface marker expression. Data are expressed as a percentage of cells that co-express CD45 and CD11b. Representative dot plots showing the CD45 and CD11b expressing cell population plus and minus LPS treatment are also shown **(Biii)**. Data are pooled samples from at least five separate experiments. ***P < 0.001 vs. untreated.

We then investigated protein levels in DAMP treated mixed glial cultures for IL-1β, IL-1α, IL-6, and CXCL1, which we have previously reported to be upregulated in response to focal ischemic injury in both the plasma and peripheral tissues, and in the brain (Chapman et al., 2009; Denes et al., 2010a; Figure 2). After 24 h NLRP3-activating DAMPs induced no increase in the protein levels of IL-1β (Figure 2Ai) or IL-1α (Figure 2Bi), whilst MSU and CPPD did induce significant increases in the production of IL-6 (Figure 2Ci) and CXCL1 (Figure 2Di). Although raised at 24 h the effects of ATP on IL-6 and CXCL1 levels were not significant, but were when we investigated the earlier time point of 4 h (Figure 2E). LPS induced a robust increase in the levels of all proteins examined (Figure 2). The production of inflammatory mediators by DAMPs alone suggest that they are capable of inducing an inflammatory response, albeit not as robust when compared to the effects of typical PAMPs such as LPS. How these DAMPs act to induce this inflammatory response is not known. None of the DAMPs tested here are reported to act as ligands for toll-like receptor (TLR). However, as mentioned in the introduction MSU can induce Syk kinase signaling (Ng et al., 2008). These DAMPs also caused some cell death within our cultures (data not shown) and so it is possible that this contributed to the effects on cytokine production observed. Microglia can upregulate CD11c and MHCII markers on the cell surface after cerebral ischemia, resulting in a phenotype resembling that of dendritic cells (Felger et al., 2010). We investigated whether PAMPs and DAMPs can directly activate an antigen-presenting phenotype in microglia. Flow cytometry showed that LPS induced an activation of cell surface CD11c and MHCII in microglia, whilst DAMPs alone had no effect (Figure 3). These data suggest that the NLRP3-inflammasome activating DAMPs investigated here, when applied alone, do not act as a priming stimulus for microglial antigen presentation.

**Figure 4:**
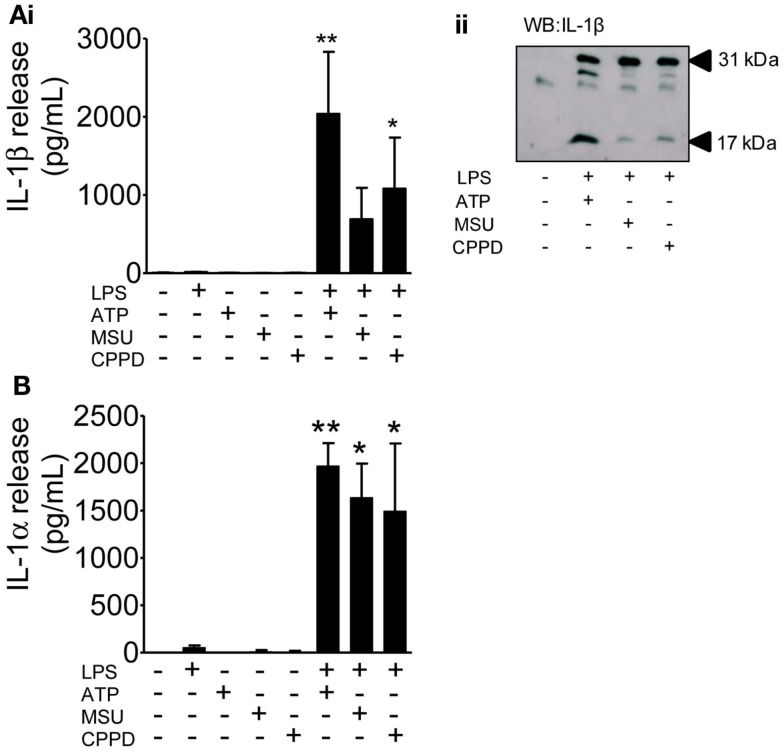
**The effects of PAMPs and DAMPs on the release of IL-1 from cultured mixed glia**. Cultured mixed glia were treated (1 h) with the NLRP3-activating DAMPs ATP (5 mM), MSU, and CPPD (both 250 μg/ml) plus and minus a 24 h priming stimulus with the PAMP LPS (1 μg/ml). Release of both IL-1β **(A)** and IL-1α **(B)** were quantified by ELISA **(Ai)**, and processing of pro- (31 kDa) to mature (17 kDa) IL-1β in PAMP and DAMP treated cells was analyzed by Western blot **(Aii)**. Data are pooled samples from at least five separate experiments. **P < 0.01, *P < 0.05 vs. untreated.

These DAMPs are known to engage the NLRP3-inflammasome and to activate caspase-1 resulting in the processing and release of IL-1β from primed cells. Although much of the literature on IL-1 secretion comes from work on peripheral macrophages, PAMP and DAMP-dependent IL-1 responses from microglia have been reported (e.g., Brough et al., 2002; Halle et al., 2008). The limited effects of these DAMPs observed so far was not due to their use at an insufficient concentration since priming of mixed glial cultures with LPS followed by treatment with ATP, MSU, or CPPD induced caspase-1 activation and the release of mature IL-1β (Figure 4A), and IL-1α (Figure 4B).

**Figure 5:**
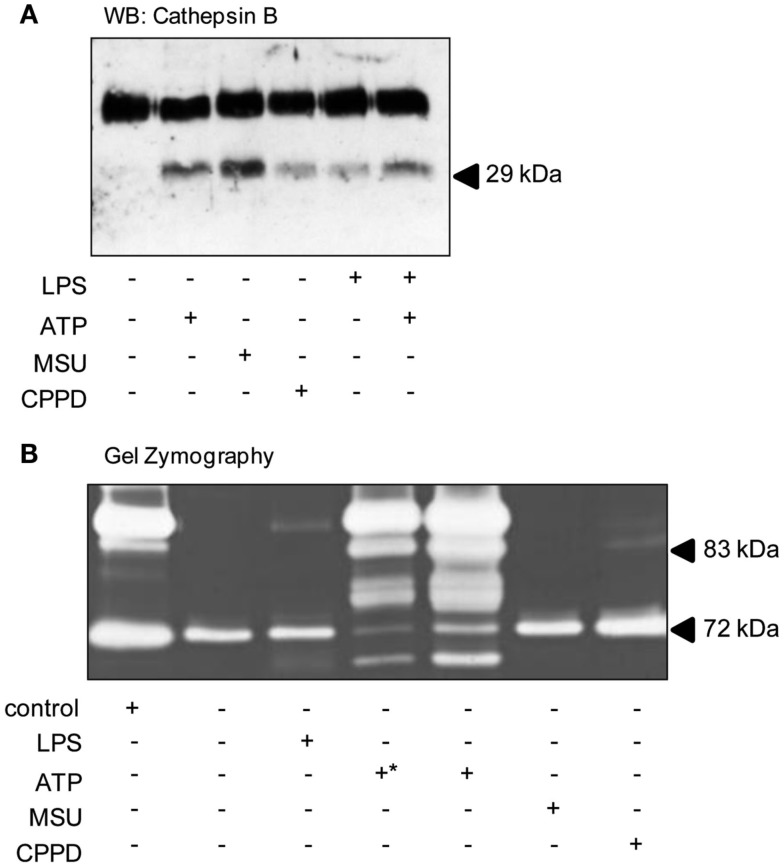
**DAMPs induce the release of proteases from cultured mixed glia**. Cultured mixed glia were treated with the NLRP3-activating DAMPs ATP (5 mM), MSU, and CPPD (both 250 μg/ml) or the PAMP LPS (1 μg/ml) for 24 h. Supernatants were analyzed for the release of mature Cathepsin B (28–30 kDa) by Western blot **(A)**. To measure the release of martix metalloproteases by zymography all samples were loaded neat onto gels except for one of the ATP-treated supernatants (+*) which was diluted 1:3 **(B)**. Blots and gels shown are representative of at least five separate experiments.

A key step during cerebral ischemia is the early breakdown of the BBB, although it is not known whether this is facilitated by the release of DAMPs from dying cells. Therefore we investigated the effects of DAMPs on the release of cathepsin B and of gelatinases which have been reported to be released following ATP-dependent activation of the P2X7 receptor in the absence of PAMP priming (Gu and Wiley, 2006; Lopez-Castejon et al., 2010), and which contribute to BBB damage after brain injury (Candelario-Jalil et al., 2009). In cultures of mixed glia ATP, MSU, CPPD, and LPS all induced the release of cathepsin B mature single chain (28–30 kDa) form (Figure 5A). Gelatin gel zymography of supernatants from cultures of mixed glia revealed that pro-MMP2 (72 kDa) was constitutively released under control conditions and that treatment of cultures with LPS, or with the DAMPs MSU or CPPD had no effect on released gelatinase activity (Figure 5B). P2X7 receptor activation in monocytes induces the release of active MMP9 (Gu and Wiley, 2006), and here in our cultures of mixed glia ATP-treatment induced a massive increase in released gelatinase activity (Figure 5B).

**Figure 6:**
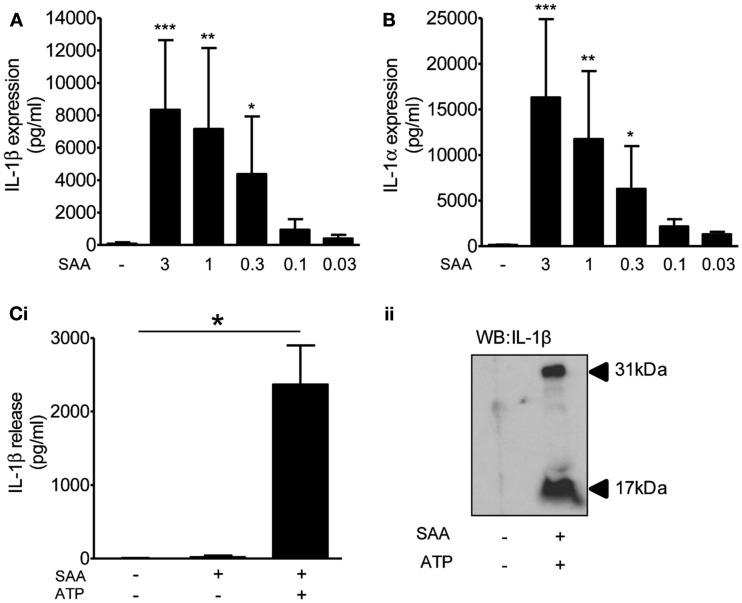
**The effects of DAMPs on the release of IL-1 from cultured mixed glia primed with serum amyloid A (SAA)**. Cultured mixed glia were treated with the indicated concentration of SAA for 24 h and the expression of IL-1β **(A)** and IL-1α **(B)** was measured in the cell lysate by ELISA. The NLRP3-activating DAMP ATP (5 mM) was added to the cultures after a 24 h priming stimulus with SAA (3 μg/ml) and released IL-1β was quantified by ELISA **(Ci)**. And processing of pro- (31 kDa) to mature (17 kDa) IL-1β was analyzed by Western blot **(Cii)**. Data are pooled samples from at least five separate experiments. ***P < 0.001, **P < 0.01, *P < 0.05 vs. untreated.

Although many DAMPs are reported to activate PRRs of the TLR family to prime inflammatory responses (Chen and Nunez, 2010), the priming stimulus for inflammasome function in the brain *in vivo* is unknown. BBB breakdown takes place early after cerebral ischemia allowing the penetration of circulating inflammatory mediators into the brain. This coincides with systemic upregulation of acute phase proteins such as SAA, which acts as an alternative acute phase protein to CRP in mice and is upregulated in plasma as early as 4 h after MCAo (McColl et al., 2007). Four hours after MCAo in the mouse there is disruption of the BBB and microglia localized to these areas of focal BBB disruption express IL-1 (Luheshi et al., 2011), suggesting that plasma derived factors could provide the priming stimulus. Moreover, SAA is reported to prime NLRP3-inflammasome dependent responses in macrophages (Ather et al., 2011; Niemi et al., 2011), and in synovial fibroblasts (Migita et al., 2012), although its effect on brain glia is unknown. Treatment of mixed glial cultures with SAA (0.03–3 μg/ml, 24 h) induced the expression of IL-1β (Figure 6A) and IL-1α (Figure 6B). These cells were effectively primed for inflammasome dependent responses as the addition of the DAMP ATP (5 mM, 1 h) induced a significant release of IL-1β (Figure 6C). These data suggest the possibility that DAMP-dependent inflammasome responses in the brain may be primed by endogenous plasma constituents.

**Figure 7:**
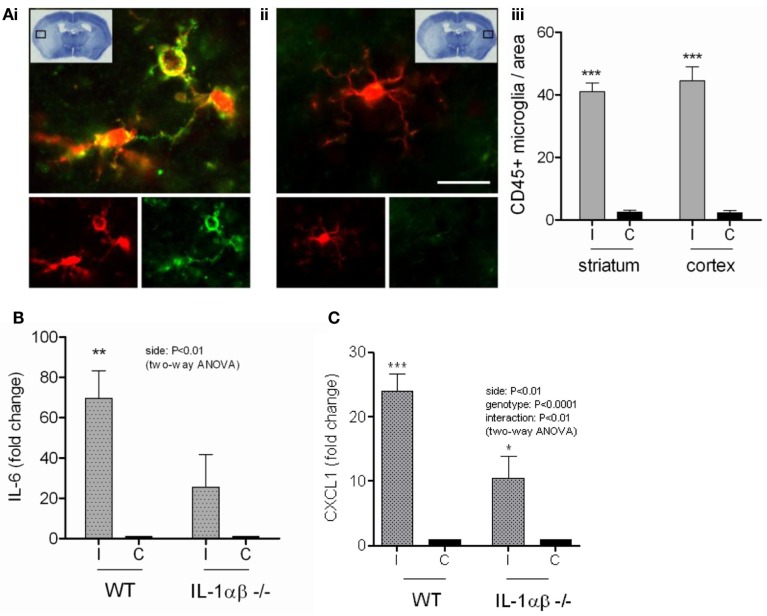
**The inflammatory response to cerebral ischemia**. Cortical cell death occurs in the ipsilateral cortex after transient middle cerebral artery occlusion (tMCAo) [insert **(Ai)**]. Microglia in this region have a significantly more activated morphology **(Ai,iii)** as shown by immunofluorescent staining for Iba1 (red) and CD45 (green), compared to those from the same region of the contralateral hemisphere **(Aii,iii)**. At the protein level *in vivo*, IL-6 **(B)** and CXCL1 **(C)** increase significantly in the ipsilateral cortex of WT mice after tMCAo. IL-6 and CXCL1 protein levels are significantly attenuated in IL-1α/β KO mice **(B,C)**. Data are expressed as fold increase over the contralateral hemisphere, are from a minimum of three independent experiments and were analyzed by two-way ANOVA followed by a Bonferonni post hoc analysis. **P < 0.01, *P < 0.05.

Since we had found that DAMPs mediated inflammation directly and also induced IL-1 release from primed glial cells *in vitro*, we aimed to investigate the effects of DAMPs on local pro-inflammatory responses induced by brain injury *in vivo* in the absence of IL-1. NLRP3-inflammasome activating DAMPs induce the release of both IL-1α and IL-1β from primed macrophages (Gross et al., 2012), and so to investigate the effects of DAMPs independently of IL-1 stimulated events we subjected WT and IL-1α/β double KO mice to transient middle cerebral artery occlusion (tMCAo) a model of experimental stroke. Thus use of the IL-1α/β double KO mice allowed us to dissect DAMP-dependent responses independently of any IL-1-mediated inflammation. Following tMCAo there was a large lesion in the ipsilateral hemisphere of mice, suggesting that glial cells in this hemisphere would be exposed to DAMPs (Figure 7Ai). Within this area there was significant microglial cell activation when compared to the contralateral hemisphere, observed by increased expression of Iba1 and CD45 (Figures 7Ai–iii). Twenty four hours after MCAo IL-6 and CXCL1 were increased in the ipsilateral hemisphere independently of the presence of IL-1 (*P* < 0.01 and 0.001, respectively, two-way ANOVA) compared to the contralateral hemisphere (Figures 7B,C). WT mice demonstrated a higher level of increase (70-fold for IL-6 and 24-fold for CXCL1) than IL-1α/β KO mice (26-fold for IL-6 and 11-fold for CXCL1), but CXCL1 was still significantly upregulated in IL-1a/b KO mice in the ipsilateral hemisphere based on Bonferroni’s *post hoc* comparison following two-way ANOVA (*P* < 0.05) and the interaction between genotype and hemispheric increase of CXCL1 was also significant (two-way ANOVA, *P* < 0.01; Figures 7B,C). IL-1β and IL-1α levels were undetectable in IL-1α/β KO mice and IL-1α displayed a significant increase after cerebral ischemia in the ipsilateral hemisphere (two-way ANOVA followed by Bonferroni’s *post hoc* test, *P* < −0.05, not shown). These data are consistent with the *in vitro* data above, suggesting that DAMPs induce a priming-independent inflammatory response, and once expression and release of IL-1 takes place, this inflammatory response is markedly augmented in the brain *in vivo*.

## Discussion

Here, we have analyzed the pro-inflammatory effects of NLRP3-activating DAMPs in the absence/presence of priming stimuli *in vitro* in cultures of mixed glial cells. As a result of our investigations we propose that in acute brain injury DAMPs can contribute to brain inflammation *via* at least three mechanisms: by directly stimulating the production of glial derived pro-inflammatory mediators, by contributing to BBB injury through the release of various proteases, and by inducing IL-1 release from primed cells. The presence of IL-1 in turn, markedly augments damage-induced inflammatory responses *in vivo*.

Inflammation is recognized as a major contributor to the worsening of acute brain injury and inhibiting IL-1 with the receptor antagonist (IL-1Ra) is protective in experimental models of stroke (Brough et al., 2011), and has shown promise as a treatment in clinical trials (Emsley et al., 2005). Early after cerebral ischemia (4 h) the related IL-1 family member IL-1α is expressed by microglia in areas of brain that will become infarct (Luheshi et al., 2011), with subsequent expression of IL-1β occurring at later time points (24 h; Denes et al., 2008). Mice in which both IL-1α and IL-1β have been deleted (IL-1α/β double KO), but not single gene KOs, have markedly reduced damage in response to MCAo (Boutin et al., 2001).

Within an ischemic tissue there will be abundant levels of DAMPs that may serve to mediate local inflammatory responses. The inflammatory actions of DAMPs are most often reported as the ability to stimulate formation of the NLRP3-inflammasome, thus activating caspase-1 and inducing the release of IL-1β (Cassel and Sutterwala, 2010). However, *in vitro* DAMPs are only reported to achieve this after the cell, typically a macrophage, has been primed with a PAMP such as LPS (Bauernfeind et al., 2009; Hornung and Latz, 2010). In monocytes and synoviocytes (Guerne et al., 1989), and osteoblast like cells (Bouchard et al., 2002) release IL-6 occurs in response to the NLRP3-inflammasome activating DAMPs MSU and CPPD in the absence of priming. However, to-date, an investigation of the pro-inflammatory effects of NLRP3-activating DAMPs in glial cells, in the absence of priming, has not taken place. Thus, in this study we sought to determine the pro-inflammatory effects of NLRP3-activating DAMPs in the presence and absence of PAMPs on inflammatory cells from the brain. We used cultures of mixed glia, composed mainly of astrocytes and microglia (Pinteaux et al., 2002). Both microglia and astrocytes represent a source of IL-1 in culture (Brough et al., 2002; Bianco et al., 2009) and from our experiments it was not possible to identify the relative contribution of each cell type to released cytokine levels. Given their similarity to macrophages, microglia are generally considered to be the major source of IL-1 in the brain after an injury (Denes et al., 2010b), yet astrocytes are also known to express IL-1β following MCAo in mice (Denes et al., 2008). It is possible that a contribution from both cellular sources drives inflammation after brain injury, although further experiments with purified cell cultures, and animal models in which solely microglia or astrocytes can produce IL-1 are required to test this.

Entirely consistent with the literature on macrophages, we found that several well characterized DAMPs (ATP, MSU, and CPPD crystals), had no effect on IL-1β levels in the absence of prior PAMP priming (Figures 1, 2, and 4). The results for IL-1α mirrored the effects of DAMPs on IL-1β, i.e., the DAMPs had no effect except when added to a PAMP-primed cell when they induced release (Figures 1, 2, and 4). In contrast, we show that DAMPs induce IL-6 and CXCL1 release from glia *in vitro* in the absence of any priming stimulus, indicating that DAMPs have a direct pro-inflammatory effect on glial cells independently of PAMP-induced signals (Figure 2).

This priming-independent inflammatory response was also observed *in vivo* after MCAo in IL-1α/β double KO mice (Figure 7). The priming stimuli for IL-1-dependent responses in the brain after a stroke are not known. There are a number of possible candidates however. For example, after disruption of the BBB plasma derived molecules known to prime IL-1β responses such as minimally oxidized LDL, and which are associated with co-morbid diseases like type II diabetes, could prime glia at the lesion site (Masters et al., 2010). Other plasma derived molecules include acute phase reactants such as SAA which has also been reported to prime NLRP3-inflammasome dependent responses in macrophages (Ather et al., 2011; Niemi et al., 2011), and we know that 4 h following MCAo in the mouse plasma SAA levels are elevated (McColl et al., 2007). We showed that SAA was capable of priming glial cultures to express IL-1α and IL-1β (Figure 6). Analogous to the PAMP priming observed with LPS, SAA alone did not induce the release of IL-1, but IL-1β release did occur after a SAA-primed culture was treated with ATP (Figure 6). In addition to these examples it is possible that one of a plethora of DAMPs reported to activate TLRs could provide the priming stimulus (Piccinini and Midwood, 2010). However, our study serves to highlight that brain inflammatory cells can respond to endogenous priming stimuli to promote IL-1-dependent inflammatory responses.

We also discovered that treatment of cultured mixed glia with DAMPs induced the release of cathepsin B, and that the DAMP ATP induced a massive release of gelatinase activity in the absence of priming (Figure 5). ATP induced cathepsin B release from non-primed macrophages results in the *in vitro* degradation of extracellular matrix, suggesting its pro-inflammatory action (Lopez-Castejon et al., 2010). That MSU and CPPD crystals also induce the release of active cathepsin B from non-PAMP-primed cells (Figure 5) suggest that this could be a common inflammatory mechanism of DAMPs. Furthermore, treatment with a cathepsin B inhibitor *in vivo* is neuroprotective following stroke (Benchoua et al., 2004). The effects of DAMP-induced release of gelatinases such as MMP9 could be twofold. MMP9 is known to disrupt the integrity of the BBB following MCAo (McColl et al., 2008), and is also known to be neurotoxic in neuroinflammatory *in vitro* models (Thornton et al., 2008). The consequence of these IL-1-independent DAMP effects would be increased production of inflammatory mediators and acute phase reactants, leukocyte recruitment, BBB disruption, and the influx of peripheral, systemic factors.

These data reveal how DAMPs induce inflammatory responses in the absence of any bacterial infection or products, and may be relevant to a range of sterile insults in addition to the model of brain injury used here. These data support a model where DAMPs released at a site of sterile injury induce the release of cytokines and proteases that are central to the establishment of an inflammatory response. DAMPs also induce the secretion of IL-1α and IL-1β from primed glia. In turn, the presence of IL-1 enhances sterile injury-induced inflammatory responses. It is not clear what primes microglia *in vivo* during ischemia but it could be one of a large number of factors, including plasma derived mediators such as SAA, and remains a subject for future investigation.

## Conflict of Interest Statement

The authors declare that the research was conducted in the absence of any commercial or financial relationships that could be construed as a potential conflict of interest.

## References

[B1] Ather J. L. Ckless K. Martin R. Foley K. L. Suratt B. T. Boyson J. E. Fitzgerald K. A. Flavell R. A. Eisenbarth S. C. Poynter M. E. (2011). Serum amyloid A activates the NLRP3 inflammasome and promotes Th17 allergic asthma in mice. J. Immunol. 187, 64–73 10.4049/jimmunol.1100500 21622869 PMC3119761

[B2] Bauernfeind F. G. Horvath G. Stutz A. Alnemri E. S. MacDonald K. Speert D. Fernandes-Alnemri T. Wu J. Monks B. G. Fitzgerald K. A. Hornung V. Latz E. (2009). Cutting edge: NF-kappaB activating pattern recognition and cytokine receptors license NLRP3 inflammasome activation by regulating NLRP3 expression. J. Immunol. 183, 787–791 10.4049/jimmunol.0901363 19570822 PMC2824855

[B3] Benchoua A. Braudeau J. Reis A. Couriaud C. Onteniente B. (2004). Activation of proinflammatory caspases by cathepsin B in focal cerebral ischemia. J. Cereb. Blood Flow Metab. 24, 1272–1279 15545923 10.1097/01.WCB.0000140272.54583.FB

[B4] Bianco F. Perrotta C. Novellino L. Francolini M. Riganti L. Menna E. Saglietti L. Schuchman E. H. Furlan R. Clementi E. Matteoli M. Verderio C. (2009). Acid sphingomyelinase activity triggers microparticle release from glial cells. EMBO J. 28, 1043–1054 10.1038/emboj.2009.110 19300439 PMC2664656

[B5] Bouchard L. de Medicis R. Lussier A. Naccache P. H. Poubelle P. E. (2002). Inflammatory microcrystals alter the functional phenotype of human osteoblast-like cells in vitro: synergism with IL-1 to overexpress cyclooxygenase-2. J. Immunol. 168, 5310–5317 11994489 10.4049/jimmunol.168.10.5310

[B6] Boutin H. LeFeuvre R. A. Horai R. Asano M. Iwakura Y. Rothwell N. J. (2001). Role of IL-1alpha and IL-1beta in ischemic brain damage. J. Neurosci. 21, 5528–5534 11466424 10.1523/JNEUROSCI.21-15-05528.2001 PMC6762680

[B7] Brough D. Le Feuvre R. A. Iwakura Y. Rothwell N. J. (2002). Purinergic (P2X7) receptor activation of microglia induces cell death via an interleukin-1-independent mechanism. Mol. Cell. Neurosci. 19, 272–280 10.1006/mcne.2001.1054 11860279

[B8] Brough D. Tyrrell P. J. Allan S. M. (2011). Regulation of interleukin-1 in acute brain injury. Trends Pharmacol. Sci. 32 617–622 10.1016/j.tips.2011.06.002 21788085

[B9] Candelario-Jalil E. Yang Y. Rosenberg G. A. (2009). Diverse roles of matrix metalloproteinases and tissue inhibitors of metalloproteinases in neuroinflammation and cerebral ischemia. Neuroscience 158, 983–994 10.1016/j.neuroscience.2008.06.025 18621108 PMC3584171

[B10] Cassel S. L. Sutterwala F. S. (2010). Sterile inflammatory responses mediated by the NLRP3 inflammasome. Eur. J. Immunol. 40, 607–611 10.1002/eji.200940207 20201012 PMC3601805

[B11] Chapman K. Z. Dale V. Q. Denes A. Bennett G. Rothwell N. J. Allan S. M. McColl B. W. (2009). A rapid and transient peripheral inflammatory response precedes brain inflammation after experimental stroke. J. Cereb. Blood Flow Metab. 29, 1764–1768 10.1038/jcbfm.2009.113 19654587

[B12] Chen G. Y. Nunez G. (2010). Sterile inflammation: sensing and reacting to damage. Nat. Rev. Immunol. 10, 826–837 10.1038/nri2873 21088683 PMC3114424

[B13] Denes A. Ferenczi S. Halasz J. Kornyei Z. Kovacs K. J. (2008). Role of CX3CR1 (fractalkine receptor) in brain damage and inflammation induced by focal cerebral ischemia in mouse. J. Cereb. Blood Flow Metab. 28, 1707–1721 10.1038/jcbfm.2008.64 18575457

[B14] Denes A. Humphreys N. Lane T. E. Grencis R. Rothwell N. (2010a). Chronic systemic infection exacerbates ischemic brain damage via a CCL5 (regulated on activation, normal T-cell expressed, and secreted)-mediated proinflammatory response in mice. J. Neurosci. 30, 10086–10095 10.1523/JNEUROSCI.1227-10.2010 20668193 PMC3044869

[B15] Denes A. Thornton P. Rothwell N. J. Allan S. M. (2010b). Inflammation and brain injury: acute cerebral ischaemia, peripheral, and central inflammation. Brain Behav. Immun. 24, 708–723 10.1016/j.bbi.2009.09.010 19770034

[B16] Dinarello C. A. (2011). Interleukin-1 in the pathogenesis and treatment of inflammatory diseases. Blood 117, 3720–3732 10.1182/blood-2010-07-273417 21304099 PMC3083294

[B17] Emsley H. C. Smith C. J. Georgiou R. F. Vail A. Hopkins S. J. Rothwell N. J. Tyrrell P. J. (2005). A randomised phase II study of interleukin-1 receptor antagonist in acute stroke patients. J. Neurol. Neurosurg. Psychiatr. 76, 1366–1372 10.1136/jnnp.2004.054882 16170078 PMC1739363

[B18] Felger J. C. Abe T. Kaunzner U. W. Gottfried-Blackmore A. Gal-Toth J. McEwen B. S. Iadecola C. Bulloch K. (2010). Brain dendritic cells in ischemic stroke: time course, activation state, and origin. Brain Behav. Immun. 24, 724–737 10.1016/j.bbi.2010.07.072 19914372 PMC2885548

[B19] Ginhoux F. Greter M. Leboeuf M. Nandi S. See P. Gokhan S. Mehler M. F. Conway S. J. Ng L. G. Stanley E. R. Samokhvalov I. M. Merad M. (2010). Fate mapping analysis reveals that adult microglia derive from primitive macrophages. Science 330, 841–845 10.1126/science.1194637 20966214 PMC3719181

[B20] Gross O. Yazdi A. S. Thomas C. J. Masin M. Heinz L. X. Guarda G. Quadroni M. Drexler S. K. Tschopp J. (2012). Inflammasome activators induce interleukin-1alpha secretion via distinct pathways with differential requirement for the protease function of caspase-1. Immunity 36, 388–400 10.1016/j.immuni.2012.01.018 22444631

[B21] Gu B. J. Wiley J. S. (2006). Rapid ATP-induced release of matrix metalloproteinase 9 is mediated by the P2X7 receptor. Blood 107, 4946–4953 10.1182/blood-2005-05-1834 16514055

[B22] Guerne P. A. Terkeltaub R. Zuraw B. Lotz M. (1989). Inflammatory microcrystals stimulate interleukin-6 production and secretion by human monocytes and synoviocytes. Arthritis Rheum. 32, 1443–1452 10.1002/anr.1780321114 2554932

[B23] Halle A. Hornung V. Petzold G. C. Stewart C. R. Monks B. G. Reinheckel T. Fitzgerald K. A. Latz E. Moore K. J. Golenbock D. T. (2008). The NALP3 inflammasome is involved in the innate immune response to amyloid-beta. Nat. Immunol. 9, 857–865 10.1038/ni.1636 18604209 PMC3101478

[B24] Hornung V. Latz E. (2010). Critical functions of priming and lysosomal damage for NLRP3 activation. Eur. J. Immunol. 40, 620–623 10.1002/eji.200940185 20201015 PMC3893565

[B25] Kleiner D. E. Stetler-Stevenson W. G. (1994). Quantitative zymography: detection of picogram quantities of gelatinases. Anal. Biochem. 218, 325–329 10.1006/abio.1994.1186 8074288

[B26] Kono H. Chen C. J. Ontiveros F. Rock K. L. (2010). Uric acid promotes an acute inflammatory response to sterile cell death in mice. J. Clin. Invest. 120, 1939–1949 10.1172/JCI40124 20501947 PMC2877935

[B27] Kool M. Willart M. A. van Nimwegen M. Bergen I. Pouliot P. Virchow J. C. Rogers N. Osorio F. Reis e Sousa C. Hammad H. Lambrecht B. N. (2011). An unexpected role for uric acid as an inducer of T helper 2 cell immunity to inhaled antigens and inflammatory mediator of allergic asthma. Immunity 34, 527–540 10.1016/j.immuni.2011.04.012 21474346

[B28] Lopez-Castejon G. Brough D. (2011). Understanding the mechanism of IL-1beta secretion. Cytokine Growth Factor Rev. 22, 189–195 10.1016/j.cytogfr.2011.10.001 22019906 PMC3714593

[B29] Lopez-Castejon G. Theaker J. Pelegrin P. Clifton A. D. Braddock M. Surprenant A. (2010). P2X7 receptor-mediated release of cathepsins from macrophages is a cytokine-independent mechanism potentially involved in joint diseases. J. Immunol. 185, 2611–2619 10.4049/jimmunol.1000436 20639492

[B30] Luheshi N. M. Kovacs K. J. Lopez-Castejon G. Brough D. Denes A. (2011). Interleukin-1alpha expression precedes IL-1beta after ischemic brain injury and is localised to areas of focal neuronal loss and penumbral tissues. J. Neuroinflammation 8, 186 10.1186/1742-2094-8-186 22206506 PMC3259068

[B31] Luheshi N. M. Rothwell N. J. Brough D. (2009). Dual functionality of interleukin-1 family cytokines: implications for anti-interleukin-1 therapy. Br. J. Pharmacol. 157, 1318–1329 10.1111/j.1476-5381.2009.00331.x 19681864 PMC2765320

[B32] Mariathasan S. Weiss D. S. Newton K. McBride J. O’Rourke K. Roose-Girma M. Lee W. P. Weinrauch Y. Monack D. M. Dixit V. M. (2006). Cryopyrin activates the inflammasome in response to toxins and ATP. Nature 440, 228–232 10.1038/nature04515 16407890

[B33] Martinon F. Petrilli V. Mayor A. Tardivel A. Tschopp J. (2006). Gout-associated uric acid crystals activate the NALP3 inflammasome. Nature 440, 237–241 10.1038/nature04516 16407889

[B34] Masters S. L. Dunne A. Subramanian S. L. Hull R. L. Tannahill G. M. Sharp F. A. Becker C. Franchi L. Yoshihara E. Chen Z. Mullooly N. Mielke L. A. Harris J. Coll R. C. Mills K. H. Mok K. H. Newsholme P. Nuñez G. Yodoi J. Kahn S. E. Lavelle E. C. O’Neill L. A. (2010). Activation of the NLRP3 inflammasome by islet amyloid polypeptide provides a mechanism for enhanced IL-1beta in type 2 diabetes. Nat. Immunol. 11, 897–904 10.1038/ni.1935 20835230 PMC3103663

[B35] McColl B. W. Rothwell N. J. Allan S. M. (2007). Systemic inflammatory stimulus potentiates the acute phase and CXC chemokine responses to experimental stroke and exacerbates brain damage via interleukin-1- and neutrophil-dependent mechanisms. J. Neurosci. 27, 4403–4412 10.1523/JNEUROSCI.5376-06.2007 17442825 PMC6672305

[B36] McColl B. W. Rothwell N. J. Allan S. M. (2008). Systemic inflammation alters the kinetics of cerebrovascular tight junction disruption after experimental stroke in mice. J. Neurosci. 28, 9451–9462 10.1523/JNEUROSCI.2674-08.2008 18799677 PMC6671112

[B37] Migita K. Koga T. Satomura K. Izumi M. Torigoshi T. Maeda Y. Izumi Y. Jiuchi Y. Miyashita T. Yamasaki S. Aiba Y. Komorimm A. Nakamura M. Motokawa S. Kawakami A. Nakamura T. Ishibashi H. (2012). Serum amyloid A triggers the mosodium urate-mediated mature interleukin-1beta production from human synovial fibroblasts. Arthritis Res. Ther. 14, R119 10.1186/ar3849 22608202 PMC3446500

[B38] Ng G. Sharma K. Ward S. M. Desrosiers M. D. Stephens L. A. Schoel W. M. Li T. Lowell C. A. Ling C. C. Amrein M. W. Shi Y. (2008). Receptor-independent, direct membrane binding leads to cell-surface lipid sorting and Syk kinase activation in dendritic cells. Immunity 29, 807–818 10.1016/j.immuni.2008.09.013 18993083 PMC2642965

[B39] Niemi K. Teirila L. Lappalainen J. Rajamaki K. Baumann M. H. Oorni K. Wolff H. Kovanen P. T. Matikainen S. Eklund K. K. (2011). Serum amyloid A activates the NLRP3 inflammasome via P2X7 receptor and a cathepsin B-sensitive pathway. J. Immunol. 186, 6119–6128 10.4049/jimmunol.1002843 21508263

[B40] Piccinini A. M. Midwood K. S. (2010). DAMPening inflammation by modulating TLR signalling. Mediators Inflamm. 2010, pii: 672395. 10.1155/2010/672395 20706656 PMC2913853

[B41] Pinteaux E. Parker L. C. Rothwell N. J. Luheshi G. N. (2002). Expression of interleukin-1 receptors and their role in interleukin-1 actions in murine microglial cells. J. Neurochem. 83, 754–763 10.1046/j.1471-4159.2002.01184.x 12421347

[B42] Schroder K. Tschopp J. (2010). The inflammasomes. Cell 140, 821–832 10.1016/j.cell.2010.01.040 20303873

[B43] Takeuchi O. Akira S. (2010). Pattern recognition receptors and inflammation. Cell 140, 805–820 10.1016/j.cell.2010.01.022 20303872

[B44] Thornton P. Pinteaux E. Allan S. M. Rothwell N. J. (2008). Matrix metalloproteinase-9 and urokinase plasminogen activator mediate interleukin-1-induced neurotoxicity. Mol. Cell. Neurosci. 37, 135–142 10.1016/j.mcn.2007.09.002 17939964

